# Expression of ezrin, CD44, and VEGF in giant cell tumor of bone and its significance

**DOI:** 10.1186/s12957-015-0579-5

**Published:** 2015-05-01

**Authors:** Jing Zhang, Jian Dong, Zuozhang Yang, Xiang Ma, Jinlei Zhang, Mei Li, Yun Chen, Yingying Ding, Kun Li, Zhiping Zhang

**Affiliations:** Bone and Soft Tissue Tumors Research Center of Yunnan Province, Department of Orthopaedics, The Third Affiliated Hospital of Kunming Medical University, Tumor Hospital of Yunnan Province, Kunming, Yunnan 650118 People’s Republic of China; Department of Pathology, The Third Affiliated Hospital of Kunming Medical University, Tumor Hospital of Yunnan Province, Kunming, Yunnan 650118 People’s Republic of China; Department of Radiology, The Third Affiliated Hospital of Kunming Medical University, Tumor Hospital of Yunnan Province, Kunming, 650118 People’s Republic China

**Keywords:** Giant cell tumor of bone, Ezrin, CD44, VEGF, Prognosis

## Abstract

**Background:**

This research aimed to study the role of ezrin, CD44, and VEGF in invasion, metastasis, recurrence, and prognosis of giant cell tumor of bone (GCTB) and its association with the clinical and pathological features of GCTB.

**Methods:**

Expression status of ezrin, CD44, and VEGF in 80 GCTB tissues and its adjacent noncancerous tissue samples were measured with immunohistochemical and Elivison staining. Their correlation with the clinical and pathologic factors was statistically analyzed by chi-square test.

**Results:**

The expression status of ezrin, CD44, and VEGF were significantly higher in GCTB tissue samples than in its adjacent noncancerous tissue samples and in GCTB at Campanacci stage III than in Campanacci stages I and II (*P* < 0.05). No significant difference was found in age and sex of the patients and locations of the tumor (*P* > 0.05). Survival analysis showed that the expression status of ezrin, CD44, VEGF, and Campanacci clinical stages of GCTB were positively associated with the survival rate of GCTB patients and negatively associated with ezrin and Campanacci stages of GCTB, indicating that ezrin, CD44, VEGF, and Campanacci clinical stages of GCTB are the independent factors for GCTB.

**Conclusions:**

Ezrin, CD44, and VEGF are over-expressed in GCTB tissue and its adjacent noncancerous tissue samples and may play an important role in the occurrence, invasion, metastasis, and recurrence of GCTB. Measurement of ezrin, CD44, and VEGF expression status may contribute to the judgment of prognosis of GCTB patients.

## Background

Giant cell tumor of bone (GCTB), a primary tumor of bone, frequently occurs in metaphysis of long bones, especially in the distal femur and proximal tibia [1,2]. It has a high recurrence rate after surgery with involvement of joints and their surrounding soft tissues and even causes serious dysfunction. Sometimes, it also incurs lung metastasis. At present, Campanacci imaging classification was an accepted classification standard of GCTBs.

GCTB is a low-grade malignant tumor with invasive, expansive, and destructive growth, and its biological behavior and prognosis are uncertain. At present, the relation between its particular gene expression and biological behavior is a hot research topic.

Ezrin is abnormally expressed in many tumor tissues and affects cellular motility, proliferation, apoptosis, and cycle and plays an important role in invasion and metastasis of tumors. In recent years, it has attracted more attention of researchers with more researches [3]. CD44 is a kind of cell adhesion factors, and changes in its expression are related to the progression, invasion, and metastasis of tumors and tumor-free survival rate of patients. VEGF is an important predisposing factor for tumor angiogenesis, promotes tumor cell proliferation, hydrolyzes basement membrane, and enhances vascular permeability through its specific binding with the receptor. Therefore, ezrin, CD44, and VEGF were used as the research targets in this study.

In this study, expression status of ezrin, CD44, and VEGF were measured with immunohistochemical technique in GCTB tissue samples, association of ezrin, CD44, and VEGF with clinical pathological characteristics and prognosis of GCTB patients, and their role in invasion, metastasis, and recurrence of GCTB was analyzed in order to provide the theoretical basis for the treatment of GCTB.

## Methods

### General data

Paraffin-embedded tumor tissue samples were taken from 80 GCTB patients who underwent operation and treatment from 1998 to 2010 in Yunnan Provincial Tumor Hospital. We have received a consent obtained from the patients and approval from the institutional review board of Tumor Hospital of Yunnan Province. Paraffin-embedded adjacent normal tissue samples collected 3 cm or farther from the tumor at operation served as controls. All samples were tested by two pathologists according to the WHO diagnostic criteria. Of the 80 patients included in this study, 45 were males and 35 were females aged 14 to 57 years (mean: 29.5 years). The tumor was located in the femur of 28 patients, in the tibia of 19 patients, in the humerus of 12 patients, in the fibula of 12 patients, and in the radius of 9 patients. Of the 80 patients, 13 were classified as Campanacci grade I, 26 as Campanacci grade II, 41 as Campanacci grade III, 49 underwent intra-lesion curettage (including 21 underwent autologous bone grafting, 15 allogeneic bone grafting, and 13 bone cement filling) and 31 underwent tumor resection (including 5 underwent allogenic bone transplantation, 23 artificial joint replacement, and 3 vascularized fibular grafting), 30 had recurrence, and 7 had pulmonary metastasis after operation (Table 1). Five patients died of pulmonary metastasis in within 2 years.

**Table 1:** **General information about the patients included in this study**

**Clinical parameter**		**Cases**
		***n***
Sex	Male	45
	Female	35
Age	≦30	33
	>30	47
Site	Femur	28
	Tibia	19
	Humerus	12
	Fibula	12
	Radius	9
Stage	I	13
	II	26
	III	41
Recurrence or metastasis	No	43
	Yes	37

The patients were followed up by telephone or E-mail for 60 months (range: 7 to 112 months). The following parameters about each patient were recorded, including the follow-up start date, tumor-free survival time of the patients, and censored value which necessitates recording the patients who failed to recover during the follow-up, died of other causes, or still had no relapse or metastasis at the end of follow-up.

### Methods

Immunophenotypes of ezrin, CD44, and VEGF were identified with the two-step immunohistochemical and Elivison staining. First, the collected tissue samples were embedded in paraffin and a volume fraction of 0.03 was obtained by dewaxing and hydrating the tissue sections which were incubated in H_2_O_2_ for 20 min in order to block the endogenous peroxidase. The antigen was repaired for 5 min in a microwave containing citrate buffer solution and washed with distilled water and PBS, respectively, for 5 min. Finally, the samples were incubated in water bath at 37°C for 2 h after drop-adding the 1st antibody dilution (with the titers of the 3 types of antibody at 1: 50) and washed 3 times with PBS (2 min at each time). The samples were then completely flushed, counterstained with hematoxylin, washed with water, dehydrated after treated with the DAB solution, mounted on slides, and observed under a microscope.

After the slides were observed under microscope, ezrin was mainly distributed in the cytoplasm as brown yellow particles, according to the intensity of staining and the number of positive cells. In accordance with Mathew’s [4] and others’ classification standards, ‘−’ indicates no expression, ‘+’ indicates that the number of expressed positive cells is less than 50% or stained lighter, and ‘+ +’ indicates that more than 50% cells are expressed and stained deep. The cells whose cytoplasm contains brown granules are positive CD44 cells. The positive cell percentage for each section was calculated in five high-power fields with (−) referring to 10% and (+) to more than 10%. VEGF was mainly distributed in cell plasma and less distributed in cell membrane. Cell plasma or membrane with brown granules was positive. The positive cell ratio of negative cases was less than 10%, while the positive cell ratio of positive cases was more than 10%.

### Statistical analysis

Statistical analysis was performed using the SPSS19.0 software package. The relation between ezrin, CD44, VEGF, and clinicopathological factors for GCTB was analyzed by **χ**^2^ test. The correlation between ezrin, CD44, and VEGF was studied by Spearman correlation analysis. Tumor-free survival curve was plotted for the survival time of patients with the 90-month follow-up data according to the different factors. In addition, Kaplan-Meier, log-rank, and multivariate Cox regression analysis showed that the expression status of ezrin, CD44, and VEGF in GCTB patients were significantly different (*P* < 0.05, *P* < 0.01). As the reported expression status of ezrin is lower in normal tissue than in GCTB tissue [5], the Campanacci stages I to III of GCTB, recurrence or metastasis of GCTB, and recurrence- and metastasis-free GCTB were compared.

## Results

### Ezrin, CD44, and VEGF immunohistochemical staining results

**Figure 1:**
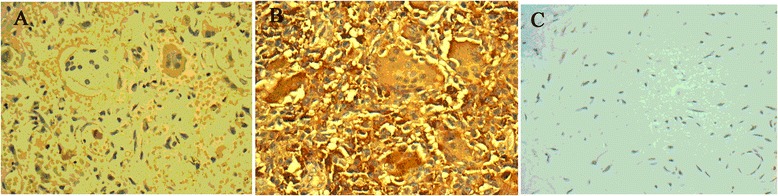
Expression of Ezrin in GCTB and normal tissues (IHC, ×400). **(A)** Low expression of ezrin in GCTB tissue (+) mainly located in the cytoplasm. **(B)** High expression of ezrin in GCTB tissue (+ +) mainly located in the cytoplasm. **(C)** No expression of ezrin in adjacent normal tissue.

Ezrin was expressed mainly in the cytoplasm as brown granules (Figure 1). Because there are few samples with expression under 20%, we choose a 50% cutoff as high expression of ezrin after dealing with statistics process. The high expression rates of ezrin were 41.2% (33/80) and 58.8% (47/80), respectively, in GCTB tissue, while its high expression rate was 11.2% (9/80) in adjacent normal tissue (**χ**^2^ = 39.670, *P* < 0.001, Table 2).

**Table 2:** **Expression of ezrin, CD44, and VEGF in different tissues**

**Sample**	**Cases**	**Ezrin**		**CD44**		**VEGF**	
	***n***	**−/+**	**++**	**−**	**+**	**−**	**+**
GCTB	80	33 (41.2%)	47 (58.8%)	50 (62.5%)	30 (37.5%)	31 (38.7%)	49 (61.3%)
Normal tissues	80	71 (88.8%)	9 (11.2%)	70 (87.5%)	10 (12.5%)	59 (73.8%)	21 (26.2%)
*P*		<0.001		0.003		<0.001	

GCTB, giant cell tumor of bone; VEGF, vascular endothelial growth factor.

**Figure 2:**
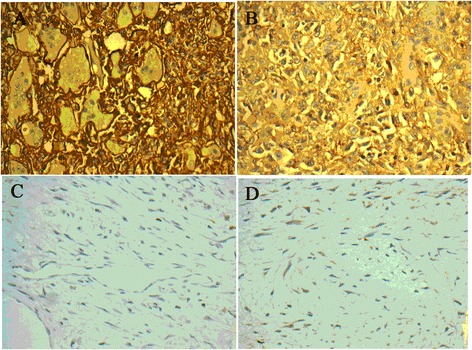
Expression of CD44 and VEGF in GCTB tissue (IHC, ×400). **(A)** CD44 was expressed as brown granules in GCTB tissue; **(B)** VEGF was expressed as brown granules in GVTB tissue; **(C)** CD44 was not expressed in adjacent normal tissues; **(D)** VEGF was not expressed in adjacent normal tissues.

CD44 was mainly expressed as brown granules in cell plasma and membrane (Figure 2A). The high expression rate of CD44 was 37.5% (30/80) and 12.5% (10/80), respectively, in GCTB tissue and its adjacent normal tissue (**χ**^2^ = 13.333, *P* = 0.003, Table 2).

The high VEGF expressions were brown granules located mainly in cell plasma and partially in the endothelial cytoplasm. The extracellular matrix was weakly expressed (Figure 2B). The VEGF high expression rate was 61.3% (49/80) in GCTB tissue and 26.2% (21/80) in adjacent normal tissues (**χ**^2^ = 19.911, *P* < 0.001, Table 2).

### Correlation between expressions levels of ezrin, CD44, and VEGF in GCTB patients

The high expression rate of ezrin, CD44, and VEGF was 58.8% (47/80), 37.5% (30/80), and 61.3% (49/80), respectively, in GCTB patients. Spearman rank correlation analysis showed that the ezrin and CD44 coefficient (*r* = 0.597, *P* < 0.001), ezrin and VEGF coefficient (*r* = 0.741, *P* < 0.001), and VEGF and CD44 coefficient (*r* = 0.616, *P* < 0.001) were positively correlated.

### Relation between ezrin, CD44, and VEGF expression in GCTB patients and their clinical data

The expression status of ezrin, CD44, and VEGF were not related with gender and age of GCTB patients and the location of GCTB (*P* > 0.05, Table 2), while it was remarkably higher in GCTB patients with recurrence and metastasis than in those without recurrence and metastasis (*P* < 0.05, Table 2). The high expression rate of ezrin, CD44, and VEGF was higher in Campanacci stages I to III of GCTB (*P* < 0.05, Table 3).

**Table 3:** **Relation between ezrin, CD44, and VEGF expressions in GCTB patients and their clinical pathological features**

**Clinical parameter**	***n***	**Ezrin**		**CD44**		**VEGF**	
		**−/+**	**++**	**−**	**++**	**−**	**++**
Sex							
Male	45	20	25	31	14	18	27
Female	35	13	22	19	16	13	22
*P*		0.510		0.181		0.795	
Age							
≤30	33	15	18	22	11	13	20
>30	47	18	29	28	19	18	29
*P*		0.522		0.519		0.921	
Site							
Femur	28	12	16	21	7	11	17
Tibia	19	9	10	12	17	8	11
Humerus	12	5	7	6	6	6	6
Fibula	12	4	8	8	4	4	8
Radius	9	3	6	3	6	2	7
*P*		0.930		0.208		0.777	
Stage							
I	13	6	5	11	2	7	6
II	26	14	12	19	7	13	13
III	41	11	30	20	21	11	30
*P*		0.007		0.009^*^		0.025	
Recurrence or metastasis							
No	43	24	19	32	11	21	22
Yes	37	9	28	18	19	10	27
*P*		0.004		0.018		0.046	

^*^Fisher’s exact test value: comparison of Campanacci stages I, II, and III. VEGF, vascular endothelial growth factor.

### Survival analysis

**Figure 3:**
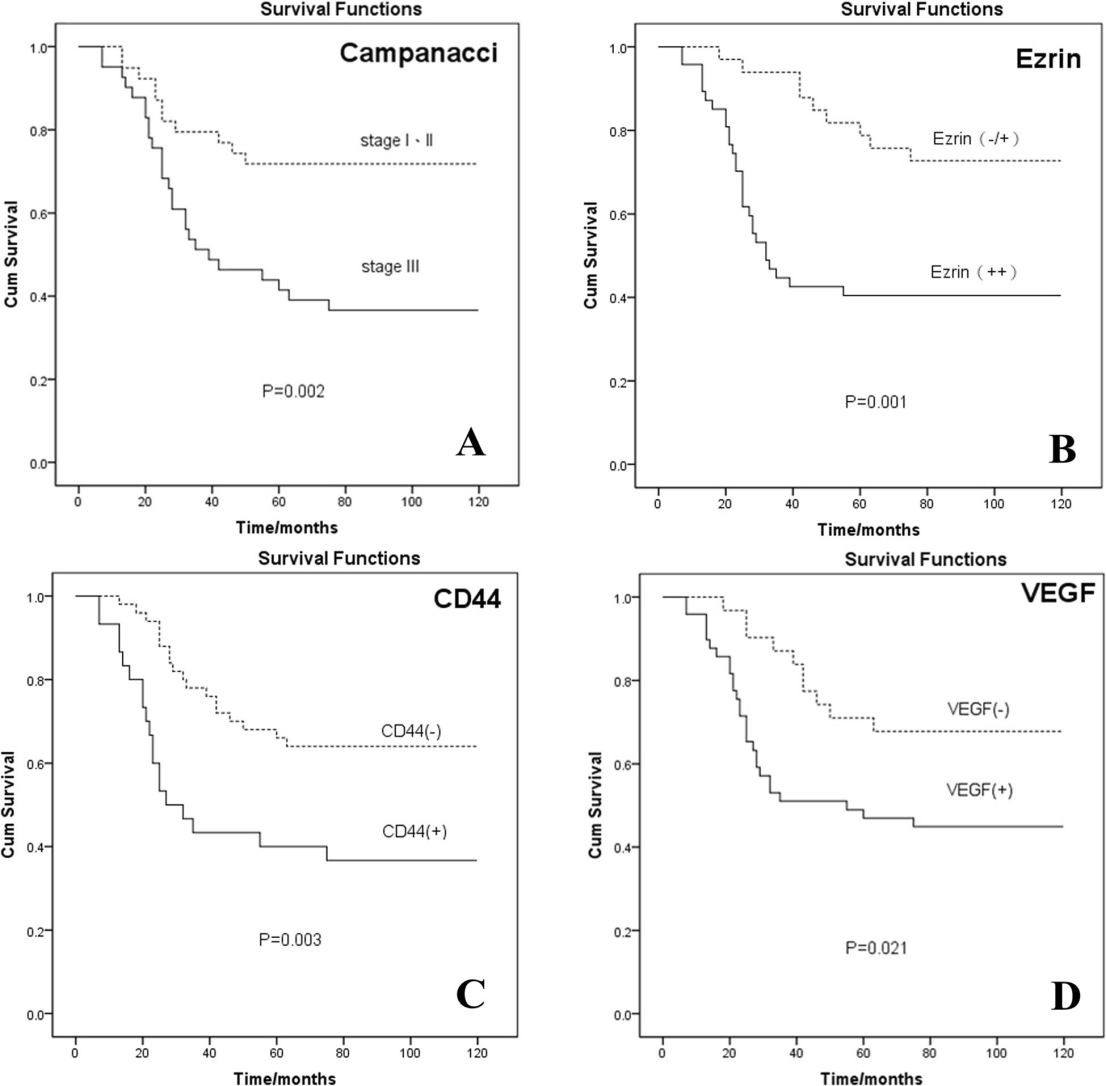
Survival curves of GCTB patients. **(A)** Campanacci stage (*P* = 0.002). **(B)** Expression of ezrin (*P* = 0.001). **(C)** Expression of CD44 (*P* = 0.003). **(D)** Expression of VEGF (*P* = 0.021). Cum, cumulative.

Kaplan-Meier analysis and log-rank comparison showed significant difference in tumor-free survival rates of male and female GCTB patients aged ≤30 or >30 years (*P* > 0.05). The tumor-free survival rates were significantly higher for patients with GCTB at Campanacci stages I and II than for those with GCTB at Campanacci stage III (*P* < 0.01, Figure 3A) while the tumor-free survival rate was obviously lower for ezrin (+ +) GCTB patients than for ezrin (−/+) GCTB patients (*P* < 0.01, Figure 3B). The tumor-free survival rate was significantly higher for CD44 (−) GCTB patients than for CD44 (+) GCTB patients (*P* < 0.01, Figure 3C) while the tumor-free survival rate was obviously lower for VEGF (+) GCTB patients than for VEGF (−) GCTB patients (*P* < 0.05, Figure 3D).

Multivariate Cox regression analysis showed that the risk ratios for ezrin, CD44, VEGF, stage, age, site, and sex were 3.027, 1.673, 0.684, 2.426, 0.612, 0.963, and 0.813, respectively. The risk ratios for ezrin and Campanacci stage were the relatively independent factors (*P* < 0.05, Table 4).

**Table 4:** **Hazard of recurrence and metastasis in association with multiple factors**

**Impact factors**	**Risk ratio**	**95% confidence interval**	***P*** **value**	**Standard error**
Ezrin	3.027	1.030 ~ 8.895	0.044	0.550
CD44	1.673	0.692 ~ 4.046	0.253	0.451
VEGF	0.684	0.220 ~ 2.127	0.512	0.579
Stage	2.426	1.140 ~ 5.160	0.021	0.385
Age	0.612	0.303 ~ 1.236	0.171	0.359
Site	0.963	0.752 ~ 1.234	0.766	0.126
Sex	0.813	0.411 ~ 1.606	0.551	0.347

VEGF, vascular endothelial growth factor.

## Discussion

Invasion, metastasis, and recurrence are major biological and clinical characteristics of GCTB and the most important factors affecting the prognosis of GCTB patients. Invasion, metastasis, and recurrence of tumor are a complex, changeable, and multistep process, which can be affected by many factors. In this study, the role of ezrin, CD44, and VEGF in biological behaviors of GCTB and their application was studied.

Ezrin, a link protein of cell membrane and cytoskeleton, can anchor actin in specific membrane, maintain cell polarity, take part in cell shape regulation and cell movement, interact with a variety of cell surface molecules (CD44, Met, etc.) [6], regulate cell-cell adhesion with cell-extracellular matrix, and promote metastasis and proliferation of tumor cells. Ezrin can regulate cell signal transduction by Rho factor, tyrosine kinase receptor, and Ras-to-MAPK pathway and plays an important role in cell movement, proliferation, apoptosis, cell cycle, invasion, and metastasis of tumor cells [7-9]. In addition, ezrin is related with micro-RNA and c-Myc [10,11] where ezrin affects invasion and metastasis of tumors.

It was reported that high ezrin expression levels play a role in promoting invasion and metastasis of esophageal carcinoma, breast cancer, ovarian cancer, liver cancer, osteosarcoma, and other common tumors [7,8,12-15]. High ezrin expression status can induce conversion of a variety of cell lines and abnormal proliferation [16]. Bruce et al. [5] analyzed the ezrin expression in more than 5,000 tumor tissue specimens, including breast cancer, lung cancer, prostate cancer, colon cancer, sarcoma, and their adjacent normal tissue samples with microarray immunohistochemical method, showing that high ezrin expression status is closely related with the pathological grade of sarcoma and recurrence of breast cancer.

Our study showed that the high ezrin expression rate was significantly higher in GCTB tissue than in its adjacent normal tissue (58.8% *vs* 11.2%, *P* < 0.05). The high ezrin expression rate gradually increased in Campanacci stages I to III of GCTB and was significantly different between Campanacci stage III and stages I to II of GCTB. The ezrin expression status was significantly higher in GCTB patients with recurrence and metastasis than in those without recurrence and metastasis, indicating that ezrin expression status is higher in GCTB patients and may influence the biological behaviors of GCTB, such as invasion, recurrence, and metastasis.

Survival analysis of the follow-up data showed that ezrin induced the GCTB biological behaviors, suggesting that recurrence of GCTB is not related with the age and gender of GCTB patients and tumor-free survival rate is significantly lower for ezrin (+ +) GCTB patients than for ezrin (−, +) GCTB patients. The median recurrence and metastasis time of GCTB patients with a high ezrin expression status was 32 months. The median recurrence and metastasis of GCTB patients with a low ezrin expression was longer than that (90 months) in our study (*P* < 0.05). Kaplan-Meier analysis and log-rank comparison showed that high ezrin expression status in GCTB patients was closely related with invasion, metastasis, and recurrence of GCTB after operation.

CD44, a receptor of hyaluronic acid and a marker of tumor stem cells, is a kind of cell adhesion factors. Changes in its expression are related to the progression, invasion, and metastasis of GCTB and the tumor-free survival rate of GCTB patients. CD44 and its ligand hyaluronan are widely distributed in human body, and its binding to hyaluronic acid caused by abnormal expression of CD44 may influence the occurrence and development of tumors [17]. CD44 is involved in cell adhesion and migration, lymphocyte activation, extracellular matrix accumulation, and metastasis of tumor cells [18]. Elliott et al. [11] showed that ezrin may play an important role in tumorigenesis by regulating cell adhesion molecules. Ezrin can directly interact with the cytoplasm of CD44 molecules, influence conformation of cytoskeletal protein and its distribution, change apoptosis of tumor cells, regulate cell-cell adhesion with extracellular matrix, and promote metastasis and proliferation of tumors. In this study, CD44 was abnormally expressed in GCTB patients. The higher the Campanacci stage was, the higher the positive CD44 expression rate was. Moreover, survival analysis showed that CD44 could affect the recurrence of GCTB, and its expression was positively related to that of ezrin, indicating that the expression of CD44 is related to the occurrence, development, and infiltration of GCTB.

Kumta et al. [19] detected the expression of VEGF in 14 GCTB patients by semiquantitative RT-PCR and immunohistochemistry, respectively, showing that multiple subtypes of VEGF, especially 121 subtypes, are highly expressed in GCTB patients and the expression of VEGF is related to the osteolytic destruction and local recurrence of GCTB. In this study, the high expression rate of VEGF was significantly higher in GCTB tissues than in its adjacent normal tissues (61.3% *vs* 26.2%). In addition, the VEGF expression status was higher in GCTB patients at Campanacci stage III with recurrence and metastasis than in those at Campanacci stages I and II with no recurrence and metastasis. The results of our study agreed with those of Kumta [19]. However, the mechanism of VEGF is not fully understood. Knowles et al. [20] showed that hypoxia can induce the expression of hypoxia-inducible factor (HIF) and regulate the expression of VEGF by autocrine and paracrine which indirectly affect the formation of osteoclasts. Matsumoto et al. [21] reported that the VEGF-Flt-1-FAK pathways consisting of VEGF and its receptor are involved in aggregation and proliferation of osteoclast precursor cells and the bone destruction. Furthermore, the expression of ezrin and VEGF in GCTB patients was positively correlated with the correlation coefficient (*r* = 0.741, *P* < 0.001) in this study. Youn [22] showed that ezrin can enhance VEGF-induced nitric oxide production by activating calpain, thereby regulating the function of endothelial cells and vascular generation, suggesting that VEGF may play a role in the recurrence and metastasis of GCTB, and interact with ezrin.

## Conclusions

In conclusion, ezrin is abnormally expressed in GCTB patients and is related with the invasion, metastasis, and recurrence of GCTB. The expression of ezrin, CD44, and VEGF is related with GCTB. Ezrin, CD44, and VEGF may play an important role in the occurrence, development, invasion, metastasis, and recurrence of GCTB and can thus be used as significant predictors for the biological behaviors and prognosis of GCTB patients. However, further Cox multivariate regression analysis revealed that only ezrin and stage were the relatively independent impact factors, implying that the role of ezrin and stage are relatively important. Invasion, metastasis, and recurrence of tumors are extremely complex, as they are not caused by one or two factors. Operation methods and ideas of doctors have certain effects on the recurrence or metastasis of GCTB [23]. Therefore, the relation between ezrin and biological behaviors of GCTB needs to be verified in more experiments. Research on ezrin, CD44, and VEGF and their correlation with tumor prognosis will help to further reveal the underlying mechanism of the invasion and metastasis of GCTB and prognosis of GCTB patients.
